# Novel Non-invasive Strategy for Spinal Neuromodulation to Control Human Locomotion

**DOI:** 10.3389/fnhum.2020.622533

**Published:** 2021-01-13

**Authors:** Tatiana Moshonkina, Alexander Grishin, Irina Bogacheva, Ruslan Gorodnichev, Alexander Ovechkin, Ricardo Siu, V. Reggie Edgerton, Yury Gerasimenko

**Affiliations:** ^1^Pavlov Institute of Physiology, Russian Academy of Sciences, St. Petersburg, Russia; ^2^Velikie Luki State Academy of Physical Education and Sports, Velikiye Luki, Russia; ^3^Kentucky Spinal Cord Injury Research Center, Frazier Rehab Institute, University of Louisville Health, University of Louisville, Louisville, KY, United States; ^4^Department of Neurological Surgery, University of Louisville, Louisville, KY, United States; ^5^Department of Neurobiology, and Neurosurgery, University of California, Los Angeles, Los Angeles, CA, United States; ^6^Institut Guttmann Hospital de Neurorehabilitació, Institut Universitari adscrit a la Universitat Autònoma de Barcelona, Barcelona, Spain; ^7^Faculty of Science, The Center for Neuroscience and Regenerative Medicine, University of Technology Sydney, Ultimo, NSW, Australia; ^8^Department of Physiology and Biophysics, University of Louisville, Louisville, KY, United States

**Keywords:** neuromodulation, paralysis, transcutaneous electrical spinal cord stimulation, humans, motor pools

## Introduction

It is well-documented that neural control of stepping and standing can be generated in mammals within the spinal neuronal networks. Having a high level of automaticity, these locomotor-related neuronal circuits can produce a “stepping” movement pattern in the absence of input from the brain and/or peripheral afferent inputs. Recent observations have provided important insight into the properties of the spinal and supraspinal circuitry that are involved in movement control. We have shown that the spinal circuitry in mice, rats, cats, and humans can be neuromodulated to regain sensorimotor function after complete paralysis (Gerasimenko et al., 2008). We have also shown that with epidural spinal cord stimulation at the lumbar level, full weight-bearing standing, and voluntary movements of the lower limb can be recovered in humans with complete paralysis (Angeli et al., 2014). Altering spinal cord excitability enables voluntary movements after chronic complete paralysis in humans. Recent breakthrough studies reported that chronically paralyzed individuals regained the over-ground walking with balance assistance through interleaved continous lumbosacral (L1-S1) epidural stimulation and task specific locomotor training (Angeli et al., 2018; Gill et al., 2018).

We have developed a novel method of non-invasive transcutaneous spinal cord stimulation (scTS) which can modulate the excitability of spinal circuitry via electrodes placed on the skin overlying the cervical, lower thoracic, lumbosacral, and coccygeal vertebrae using a special form of electrical pulses delivered at a high frequency (Gerasimenko et al., 2015a). This methodology was able to neuromodulate the spinal locomotor networks such that involuntary stepping-like movements were induced in non-injured subjects when their legs were placed in a “gravity-neutral” apparatus (Gerasimenko et al., 2015b). In addition, our initial results with scTS show that this strategy can facilitate individuals having motor complete paralysis to generate rhythmic stepping patterns and non-repetitive voluntary movements (Gerasimenko Y. P. et al., 2015). The novel finding in this and ongoing studies is that specifically configured multisite stimulation can produce a more robust response when compared to the single site stimulation. Based on these findings, we developed a three-by-three multielectrode transcutaneous array that allows multiple sites to be modulated, thus, providing subject-specific options for controlling posture and locomotor behavior (Gerasimenko et al., 2015a). We observed that the effectiveness of inducing of involuntary stepping movements in a non-injured subject with legs placed in a “gravity-neutral” position during spinal cord stimulation at one level (T11) with 3 interconnected electrodes (A,B,C) located at midline (B) and laterally (A and C) to the spinal cord vs. stimulation at 2 levels (T11 + L1) with electrodes (T11-ABC) + (L1-ABC) was different. The amplitude of knee displacement and surface electromyographic (sEMG) activity of leg muscles were significantly higher with multi-site stimulation at 2 levels than at one level (Gerasimenko et al., 2015b). Our preliminary data reveal that use of the multielectrode surface array can fine-tune the control of the locomotor behavior. Here we introduce a new strategy of spinal neuromodulation using the continuous stimulation of locomotor circuitry and the rhythmic stimulation of motor pools.

## Combined Activation of Locomotor Circuitry and Motor Pools

This study is based on the concept of differential modulation of neuronal networks projecting to specific interneurons that coordinate the levels of recruitment of different combinations of motor pools throughout a step cycle. A novel approach of spatiotemporal spinal stimulation through rhythmic scTS coupled with continuous scTS to promote locomotor neural plasticity by activating regional spinal networks in a manner similar to that observed during normal gait has been suggested. The general idea is the use of continuous spinal stimulation to activate the locomotor networks in combination with rhythmic targeted activation of flexor and extensor motor pools of leg muscles in different phases of step cycle to further enable a stepping-like behavior. Recently it was shown that spatiotemporal epidural stimulation of flexor and extensor motor pools of left and right legs can facilitate swing or stance phases during the stepping cycle in chronic motor incomplete. Severely paralyzed (unable to walk over-ground) individuals recovered over-ground walking with balance assist (Wagner et al., 2018).

**Figure 1:**
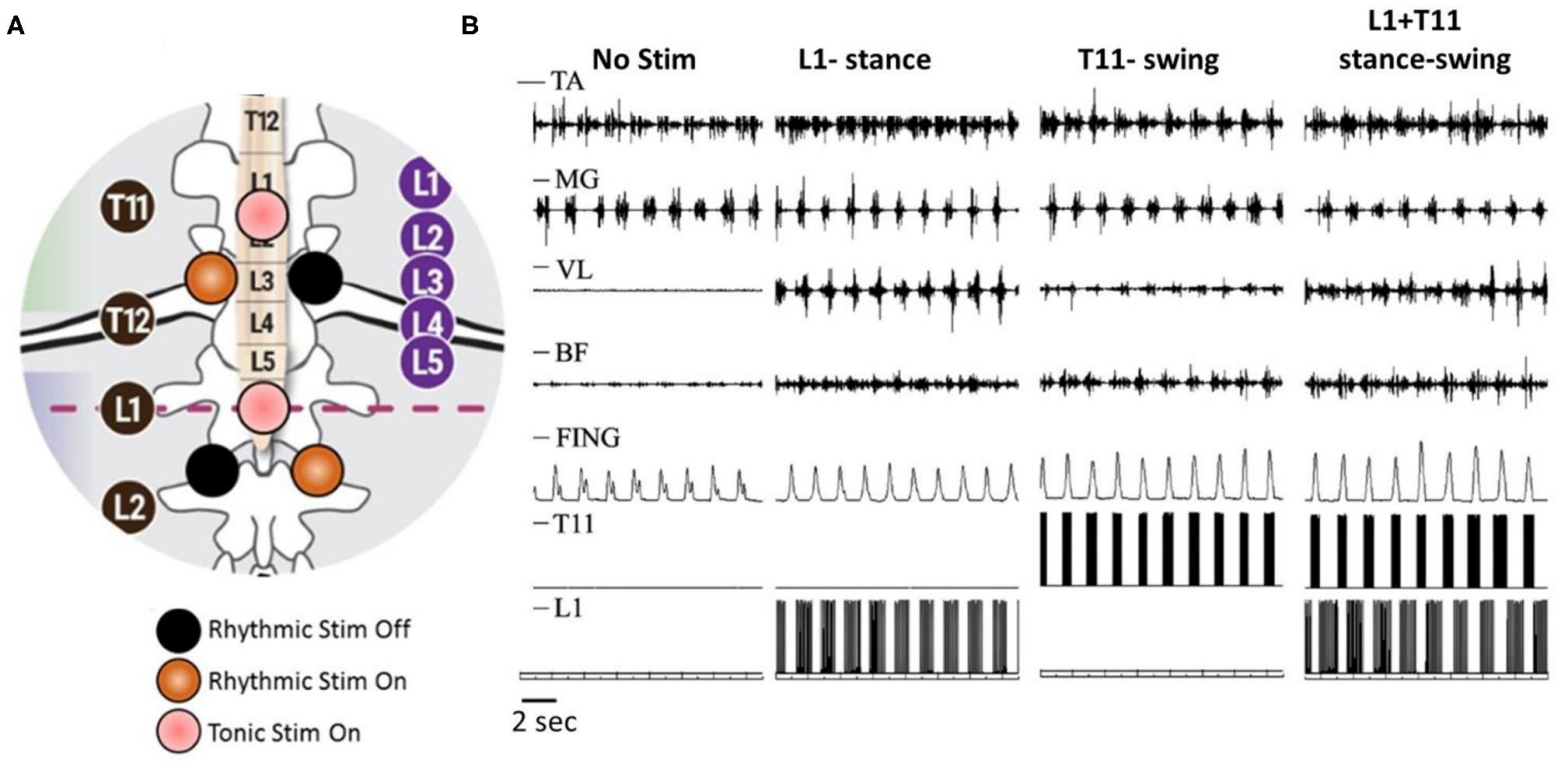
Rhythmic and continues spinal cord transcutaneous stimulation paradigm. **(A)** Schematic localization of stimulation electrodes. Midline electrodes at T11 and L1 generate continuous stimulation at 30 Hz. Lateral electrode at T11 generate rhythmic stimulation at 30 Hz during swing phase and lateral electrode at L1 generate rhythmic stimulation at 15 Hz during stance phase. **(B)** Surface EMG activity of distal [tibialis ant. (TA) and medial gastrocnemius (MG)] and proximal [vastus lat. (VL) and biceps femoris (BF)] muscles as well as displacement of terminal poit (Fing) during walking on the treadmill without stimulation, with rhythmic stimulation of L1 at stance phase, with rhythmic stimulation of T11 at swing phase, and combined L1 and T11 stimulation applied to stance and swing phase, correspondingly.

Our experimental paradigm included rhythmic scTS with two cathodes at each T11 and L1 delivered 2.5 cm lateral to the midline of the spine, and continuous scTS at T11 or L1 applied along the midline between the spinous processes (Figure 1A). ScTS was carried out with the electrical stimulator BioStim-5 (Cosyma INC). Stimulation was delivered using 2.5-cm-diameter round gel adhesive electrodes (Syrtenty Premium TENS) as cathodes, and two interconnected 5 × 9 cm^2^ self-adhesive electrodes (Axelgaard, ValuTrode Cloth) placed over the iliac crests as anodes. The stimulation waveform consisted of monophasic rectangular 1.0-ms pulses at a frequency of 15 and 30 Hz, each pulse filled with a carrier frequency of 5 kHz.

In non-injured subjects (*N* = 6, 23.7 ± 2.3 years), stance and swing phases were detected by sensors-gyroscopes. Initiation of hip extension was the trigger for activation of extensor pools (L1) during stance phase, whereas the initiation of hip flexion was triggered for activation of flexor motor pools (T11) during swing phase (Grishin et al., 2020). During walking on treadmill, stimulation applied unilaterally at L1 during the stance phase with a frequency of 15 Hz increased the amplitude of movements in the hip joint, and significantly increased sEMG activity of the extensor muscles of the thigh and flexor muscles of the shank. Meanwhile, unilateral stimulation at T11 with a frequency of 30 Hz applied during the swing phase increased walking speed due to reduction of the stance phase duration. Additionally, the stimulation induced an increase in the amplitude of movements in the hip joint and the lifting of the knee as well as foot elevation. This was accompanied by an increase of sEMG activity in BF and TA muscles (Figure 1B). Combined spatiotemporal stimulation at L1 and T11 applied to stance and swing phases, correspondingly did not cause a change in the duration of the stepping cycle and the phases of the step, however, it changed the kinematic characteristics of movements. In the stance phase, the amplitude of movements in the hip joint increased. The amplitude of movements in the hip joint increased in the swing phase as well, but it also increased the lifting of the knee and foot elevation (Figure 1B). Additional continuous stimulation applied along the midline at T11 with frequency of 30 Hz facilitated the effect of rhythmic spatiotemporal stimulation. Thus, the data obtained shows that specifically configured multi-site scTS is able to selectively facilitate the activation of the motor pools of the lower extremities and control their activity to regulate the phases of the stepping cycle.

## Discussion

Our experience with multi-electrode epidural stimulation in mice, rats, and humans (Gerasimenko et al., 2008; Harkema et al., 2011; Angeli et al., 2014) indicate the potential to regain overground load-bearing stepping, as well as voluntary control of lower limb movements, using non-invasive neuromodulatory strategies. The main issue is to determine the potential of scTS applied to different sites of the spinal cord can provide spatiotemporal specificity of the locomotor network in a way that facilitates phase-specific flexor-extensor motor pool populations. Early, we demonstrated that transcutaneous electrical stimulation of rostral and caudal areas of lumbosacral enlargement resulted in a selective topographical recruitment of proximal and distal leg muscles based on their threshold intensity, maximal slope of the recruitment curves, and plateau point intensity and magnitude (Sayenko et al., 2018). Our data are generally consistent with previous reports and myotomal maps of the spinal cord and lumbosacral roots (Stewart, 1992; Ivanenko et al., 2006). It is well-known that the lumbosacral enlargement not only contains motor neuron pools projecting to proximal and distal leg muscles, but also encompasses neuronal networks controlling locomotion and standing. We demonstrated that epidural stimulation of the spinal rostral segments (L2) is more effective for inducing rhythmic movements, whereas stimulation of more caudal segments (S1–S2) allows for greater postural control (Angeli et al., 2014). During combined locomotor-specific scTS over the T11 at 30 Hz, with the postural-specific scTS over the L1 at 15 Hz, we observed the interplay of various stimulation characteristics in generation of continuous and alternating weight-bearing, and facilitation of body-weight transitions allowing effective stepping motions to be performed (Sayenko et al., 2018). These results suggest that stimulation of multiple spinal sites related to postural and locomotor circuitries activation might be complementary and thus can be a viable strategy to facilitate more effective stepping-like and postural movements.

Given the numerous observations of the different sources and techniques to gain access to the neurons that generate locomotor rhythms, it is not surprising that modulation of these neurons, commonly referred to as the locomotor central pattern generator, can be modulated electrically to different physiological states from multiple spinal segments, as well as multiple supraspinal sites. Although the rostral lumbar segments generate more robust pattern generation, neuromodulatory effects of stimulating at S1 was not expected to give the enhancement of the locomotor rhythms derived from the fictive locomotor patterns observed in the rodent neonatal preparation as reported in series of experiments by Lev-Tov and colleagues (Lev-Tov et al., 2000). For example, changes in the endogenous levels of cholinergic components in the sacral area with ascending projections via the ventral funiculus (Finkel et al., 2014) and Alpha-1 adrenoceptor agonists in this region have been observed to modulate the fictive locomotor output (Gabbay and Lev-Tov, 2004).

We have also shown that the rostral lumbar segments are the key controllers of hindlimb locomotor rhythmicity in the adult spinal rat (Gerasimenko et al., 2019). We observed that the rats with spinal cord transections at T8 and S1 remained capable of generating coordinated hindlimb locomotion when receiving epidural stimulation at L2. In contrast, minimal locomotion was observed when S1 stimulation was delivered after spinal cord transections at T8 and L2. These findings are compatible with work of others demonstrating the critical role of commissural neurons located in rostral lumbar segments supporting locomotor rhythm generation in response to bulbospinal activation of locomotion *in vitro* neonatal rat spinal cord (Cowley et al., 2009).

Shik hypothesized the role of propriospinal system in initiation of locomotion (Shik, 1997). It has been demonstrated that microstimulation of stepping strip in the dorsolateral funiculus (DLF) elicited stepping movements in mesencephalic cats (Kazennikov et al., 1983). According to these authors, neurons responding to stimulation of the stepping strip send their axons into the ventrolateral funiculus (VLF) near the gray matter. It has been suggested that excited fibers of DLF can activate hindlimb stepping indirectly through DLF -VLF propriospinal loops (Shik, 1997). Our data are consistent with suggestion about activation the locomotor-related neuronal network through DLF. We have demonstrated that after chronic local lesion of the dorsolateral column it was impossible to induce the locomotor activity in the cat by epidural stimulation of the spinal cord (Gerasimenko et al., 2002). Recently, neurotechnology that targets specific motor pools for restoration of walking in humans with spinal cord injury (SCI) was demonstrated. The authors delivered spatiotemporal epidural stimulation to specific flexor/extensor motor pools during specific phases of the locomotor cycle. Using this targeted neurotechnology, paralyzed chronic motor incomplete individuals were able to walk over-ground with balance assist (Wagner et al., 2018). The technology we describe here is able to target these motor pools similarly to restore walking without the need of surgical implantation.

Here we demonstrated that non-invasive preferential activation of spinal structures at specific segments is possible. According to the results presented on the Figure 1B, it is clear that lateral stimulation at T11 engaged the neural circuits controlling flexion, whereas lateral stimulation at L1 primarily recruited the circuits controlling extension during stepping. Spatiotemporal T11+L1 stimulation enhanced generation of motor patterns and enabled control of leg movements. We suggest that this non-invasive technology could be effective for neuromuscular control of postural and locomotor function in post-stroke subjects and in individuals with SCI.

## Author Contributions

The concept and design of this study were developed by YG. TM, AG, IB, RG, RS, and AO were involved in data acquisition, analysis, and interpretation. The manuscript draft written by YG and TM was critically revised and approved by all authors.

## Conflict of Interest

YG researcher on the study team, hold shareholder interest in NeuroRecovery Technologies and Cosyma. He holds certain inventorship rights on intellectual property licensed by the regents of the University of California to NeuroRecovery Technologies and its subsidiaries. VE holds shareholder interest and certain inventor rights in NeuroRecovery Technologies and SpineX and holds certain inventorship rights on intellectual property licensed by The Regents of the University of California to NeuroRecovery Technologies and its subsidiaries. The remaining authors declare that the research was conducted in the absence of any commercial or financial relationships that could be construed as a potential conflict of interest.
